# Professional Fulfilment in Pharmacy: A Cross-Sectional Survey of Pharmacists in 17 European Countries

**DOI:** 10.3390/pharmacy14030073

**Published:** 2026-05-14

**Authors:** Katarina Fehir Šola, Slaven Falamić, Maja Ortner Hadžiabdić, Piotr Merks

**Affiliations:** 1 Pharmacy Bjelovar, 43000 Bjelovar, Croatia; 2 Faculty of Medicine, University of Josip Juraj Strossmayer Osijek, 31000 Osijek, Croatia; sfalamic@mefos.hr; 3 European Association of Employed Pharmacists, Spittelgasse 21/3, 1090 Viena, Austria; 4 Pharmacy Popović, 31551 Belišće, Croatia; 5 Faculty of Pharmacy and Biochemistry, University of Zagreb, 10000 Zagreb, Croatia; mortner@pharma.hr; 6 Department of Pharmacology and Clinical Pharmacology, Faculty of Medicine, Collegium Medicum Cardinal Stefan Wyszyński University, 01-815 Warsaw, Poland; p.merks@uksw.edu.pl

**Keywords:** job satisfaction, pharmacists, professional perception, workforce wellbeing, Europe

## Abstract

**Background/Objectives**: Pharmacists play an essential role in healthcare delivery across Europe, yet growing professional demands, organisational constraints, and evolving practice models may negatively affect job satisfaction and professional fulfilment. This study aimed to evaluate job satisfaction and professional perception among pharmacists across multiple European countries and to identify sociodemographic and workplace-related factors associated with these outcomes. **Methods**: A cross-sectional, web-based survey was conducted between October 2023 and January 2024 among licensed pharmacists from 17 European countries. Eligible participants were pharmacists employed in community pharmacies, hospitals, clinical pharmacy services, or the pharmaceutical industry. The questionnaire, developed and administered in English, collected sociodemographic and professional data and included two composite measures: the Job Satisfaction Scale (12 items) and the Pharmacist Professional Perception Scale (6 items). Responses were recorded using 5-point Likert scales. Descriptive statistics and inferential analyses were performed using SPSS version 27.0. **Results**: A total of 789 pharmacists participated (median age 40 years; 80.1% female). The mean job satisfaction score was 3.26 (SD 0.88), with the lowest scores related to staffing adequacy and salary, and the highest to collegial relationships. The mean professional perception score was 3.08 (SD 0.81), indicating moderate perceived professional recognition. Significant associations were identified between both scales and workplace setting, income level, employment status, geographical region, education, and professional experience (*p* < 0.05). **Conclusions**: In this multi-country convenience sample, pharmacists reported moderate levels of job satisfaction and professional perception, with variation across workplace and sociodemographic factors. These findings should be interpreted cautiously, as the sample is not representative of all European pharmacists; however, they suggest that staffing conditions, remuneration, professional recognition, and career development opportunities may be relevant areas for further investigation and policy attention.

## 1. Introduction

According to Eurostat estimates, in the European Union in 2021, a total of 1.4 million healthcare professionals, including pharmacists, dentists, and physiotherapists, were employed [1]. The ownership structure under which primary pharmacists work in Europe is diverse. European pharmacy practice varies widely beyond ownership models, especially in scope of practice, reimbursement methods, and workforce regulations, and the cultural framing of professional roles. This diversity leads to multiple national and regional pharmacy cultures instead of a unified European model. Therefore, cross-country comparisons should be understood as reflecting each country’s unique context, not as representative of Europe as a whole.

With rapid technological advances and legislative changes, pharmacists face significant challenges in their workplaces. Pharmacists are crucial to healthcare delivery, providing essential services such as medication management, clinical consultation, and patient education. Despite their vital role, increasing evidence highlights ongoing issues with pharmacists’ professional satisfaction, particularly regarding recognition, role clarity, and the alignment between training and practice [2].

Job dissatisfaction among pharmacists can directly endanger patients by increasing medication dispensing errors [3]. Many factors related to healthcare professionals’ satisfaction, such as workload, work environment, opportunities for professional development, salary, and recognition, have been identified in the literature [4,5,6,7,8,9]. According to meta-analyses, job satisfaction influences the decision to leave a pharmacist’s workplace [10]. At the core of these issues is the concept of professional identity—how pharmacists perceive their role and legitimacy within the healthcare system. Professional identity has been described as a vital element affecting responsiveness to practice innovations and professional fulfilment [11]. However, the literature also shows that many pharmacists experience ambiguity or conflict in that identity, which can undermine role satisfaction and adaptability [12].

Positive public perception of pharmacists, their role in the healthcare system, and collaboration with physicians and other healthcare professionals can enhance satisfaction.

Despite the evolution of pharmacy practice, medical students are not sufficiently informed about the potential roles of pharmacists and may not support the management of chronic patients. Some studies on public perception show that patients see pharmacists merely as salespersons [13].

Due to the rapid advances in pharmacy practice, pharmacists’ self-perception and roles have undergone significant evolution. Earlier studies indicated that pharmacists primarily viewed themselves as medication dispensers rather than patient-centred healthcare providers. Despite considerable progress in practice, challenges and dissatisfaction in delivering pharmaceutical care persist. Pharmacists often do not see themselves as clinicians, with key identity themes including Clinician, Dispenser, Businessperson, Patient Counsellor, and Physician Supporter [5].

However, a deeper understanding of how these factors manifest across different European countries, considering the unique aspects of their healthcare systems, regulatory frameworks, and cultural differences, is lacking.

To address these knowledge gaps, this study conducts a comprehensive examination of pharmacist satisfaction and self-perception across multiple European countries, establishing the foundation for recommendations to improve working conditions and promote positive professional experiences within this vital healthcare profession.

## 2. Materials and Methods

### 2.1. Survey Design and Participants

This international, web-based cross-sectional survey targeted licensed pharmacists across Europe. Eligible participants included healthcare professionals employed across various healthcare settings, including community pharmacies, hospitals, clinical pharmacy services, and the pharmaceutical industry. Pharmacists not actively practising were excluded. The survey received 789 responses from pharmacists in 17 European countries. Of the 789 respondents, 37.3% were employed on a part-time basis, ensuring representation of both full-time and part-time pharmacy practitioners across the surveyed jurisdictions. Within EU/EEA jurisdictions, pharmacist education and recognition of professional qualifications are broadly shaped by Directive 2005/36/EC, which requires at least five years of university-level pharmacy education and training. However, several participating countries fall outside this framework, and national registration procedures and degree nomenclature vary across jurisdictions. Therefore, the education variable collected in this study should not be interpreted as a direct proxy for national licensure requirements. The European Association of Employed Community Pharmacists (EPhEU) distributed the invitation link through their mailing lists and newsletters, with further sharing by partner national organisations. This method represents a convenience sampling approach, targeting pharmacists with access to these professional networks. Since the invitation was sent via external mailing lists and shared within networks, it is not possible to accurately determine the total number of pharmacists who received or saw the survey link. As a result, the study lacks a definitive sampling frame and cannot specify an exact response rate.

Participation was voluntary, unpaid, and anonymous. To maintain data quality, only one response per device was allowed, and duplicate IP entries were automatically blocked.

### 2.2. Instrumentation

The survey collected sociodemographic data alongside information on job satisfaction, workplace environment, perceptions of organisational policies, and professional recognition. The questionnaire items were systematically arranged and subsequently analysed using two composite measures: the Job Satisfaction Scale (12 items) and the Pharmacist Professional Perception Scale (6 items).

The instrument was collaboratively developed by the European Association of Employed Pharmacists, the Faculty of Pharmacy and Biochemistry (University of Zagreb, Croatia), and the Collegium Medicum, Cardinal Stefan Wyszyński University (Warsaw, Poland).

Instrument development was conducted through a multi-stage process:

1. Content validation was carried out through a focus group of five practising pharmacists not involved in the instrument’s design, leading to modifications of five questions and the removal of one item.
2. Pre-testing involving 20 pharmacists evaluated clarity, understanding, and reliability, resulting in minor wording adjustments.
3. Pilot testing confirmed internal consistency (Cronbach’s α = 0.906 for the Job Satisfaction Scale; 0.802 for the Pharmacist Professional Perception Scale).

Responses were gathered using 5-point Likert scales (1 = strongly disagree to 5 = strongly agree). Higher scores reflected greater satisfaction or recognition.

The questionnaire was developed and administered in English, which served as the working language of the collaborating organisations and enabled the use of a single instrument across countries. 

For the purposes of this study, ‘specialisation’ was defined as any formally recognised postgraduate qualification, advanced clinical title, or officially designated area of specialised pharmacy practice obtained beyond the initial licensure degree. Respondents were asked to self-report whether they held such a specialisation. It is acknowledged that the concept and formal recognition of pharmacy specialisation is not harmonised across the 17 participating countries, and that this term may have been interpreted differently by respondents from different national regulatory and professional contexts. Findings relating to specialisation should therefore be interpreted with this caveat in mind.

Monthly personal income was collected as self-reported net income using predefined categorical bands (less than 1000 euros; 1000–2000 euros; 2000–3000 euros; more than 3000 euros). Net income was selected because taxation systems differ substantially across participating countries. No adjustment was made for purchasing power parity or cost-of-living differences across national contexts.

### 2.3. Data Collection

Data was collected from 1 October 2023 to 31 January 2024, via Google Forms, a secure, GDPR-compliant platform. Participants could not revisit prior screens after completing items, but each page required confirmation to proceed. Invitations to the survey highlighted its purpose, voluntary participation, and confidentiality of responses. No personal identifiers were collected. An introductory letter accompanied the survey, explaining the objectives, estimated completion time (approximately five minutes, based on pilot testing), and contact details for the investigators. Actual individual completion times were not recorded during the main data collection phase and therefore cannot be reported.

### 2.4. Data Analysis

Descriptive statistics were used to summarise participant characteristics and item- and scale-level results. The internal consistency of the two composite measures was assessed using Cronbach’s alpha coefficient (0.906 for the Job Satisfaction Scale; 0.802 for the Pharmacist Professional Perception Scale). Statistical analyses were conducted using SPSS Version 27.0. Categorical variables are presented as frequencies and percentages, while continuous variables are reported as means and standard deviations or medians and interquartile ranges, as appropriate. Because the main outcome measures were based on Likert-scale data and several comparison groups were unequal in size, non-parametric tests were used for group comparisons. Differences between two groups were assessed using the Mann–Whitney U test, while comparisons involving three or more groups were performed using the Kruskal–Wallis test. Statistical significance was set at *p* < 0.05. For analytical purposes, some variables were grouped into broader categories to improve interpretability and ensure sufficient subgroup sizes. Age and years of professional experience were analysed in categories, and countries were also grouped by geographical region. This regional grouping was introduced to enable more robust comparisons given the uneven distribution of respondents across countries. Specifically, countries were grouped into Western Europe (Austria, France, Germany, Malta, Norway, Portugal, Switzerland, and the United Kingdom) and Eastern Europe (Bosnia and Herzegovina, Bulgaria, Croatia, Kosovo, Montenegro, Poland, Romania, Serbia, and Turkey). This regional categorisation was used as a pragmatic analytical approach and should be interpreted cautiously, as it does not capture the full heterogeneity of pharmacy practice across individual countries.

To minimise bias, responses were anonymised and the survey included attention checks to ensure data quality.

### 2.5. Ethical Considerations

The study received ethical approval from the Ethics Committee of the European Association of Employed Community Pharmacists (EPhEU) (Approval No. 1-12/2022, granted on 4 December 2022). All procedures adhered to the Declaration of Helsinki and EU General Data Protection Regulation (GDPR).

Participation was voluntary, and informed consent was obtained electronically before survey initiation. By clicking “I agree” and proceeding to the first survey item, participants confirmed consent. Responses were stored securely on encrypted servers, with access restricted to the research team.

## 3. Results

### 3.1. Participants Characteristics

The sample comprised 789 pharmacists from 17 European countries, of whom 80.1% were female. The median age was 40 years (range 20–75), and the median work experience was 13.5 years (IQR 6–22). Most participants worked in community pharmacies (83.8%), mainly in independent and large-chain settings, while fewer worked in hospitals or industry. Most participants (65.9%) were from Western Europe, with 34.1% from Eastern Europe. With respect to employment status, part-time pharmacists constituted 37.3% of the total sample. Notably, part-time employment was significantly associated with both outcome measures: part-time pharmacists reported higher job satisfaction (mean 3.35 ± 0.82 vs. 3.21 ± 0.90; *p* = 0.046) and substantially higher Pharmacist Professional Perception scores (mean 3.34 ± 0.66 vs. 2.93 ± 0.85; *p* < 0.001) compared to full-time colleagues, a finding discussed further in Section 4.

A comprehensive breakdown of sociodemographic characteristics is outlined in Table 1.

### 3.2. Job Satisfaction and Pharmacist Professional Perception Scores

To enhance the communicative value of the item-level data, Table 2 presents the mean scores (± SD) for each of the twelve individual Job Satisfaction Scale items, displayed as a ranked horizontal bar chart. This visualisation clearly delineates the within-scale heterogeneity, most strikingly illustrated by the contrast between the lowest-scoring item—staffing adequacy (‘Staffing is adequate; enough employees are hired to cover the workload in the pharmacy’; mean 2.72, SD 1.38)—and the highest-scoring item—collegial connection (‘I feel connected to my coworkers’; mean 3.84, SD 1.08). Items related to salary appropriateness (mean 2.98), talent utilisation (mean 2.83), and career commitment (‘Knowing what I know now, I would still choose pharmacy’; mean 3.00) clustered at the lower end of the scale, whilst items pertaining to temporal engagement (‘The time goes by quickly while I am at work’; mean 3.77) and working environment (mean 3.57) scored comparatively higher.

Table 3 presents the mean scores (±SD) for each of the six Pharmacist Professional Perception Scale items in ranked horizontal bar chart format. The item-level distribution reveals considerable heterogeneity within this scale. The highest-scoring item—‘Pharmacists are an essential part of the health system’ (mean 4.36, SD 1.02)—stands in marked contrast to the lowest-scoring item ‘A lay person is knowledgeable about the level of education of pharmacists’ (mean 2.40, SD 1.09)—a difference of nearly two full scale points. Items reflecting systemic recognition (‘The health system I work in recognises pharmacists’ competencies’; mean 2.74), regulatory satisfaction (mean 2.91), and interprofessional collaboration with nurses and physicians (mean 2.90) similarly scored below the scale midpoint, collectively indicating that whilst pharmacists perceive their systemic importance in abstract terms, this perception is not mirrored by concrete professional recognition within healthcare structures or by lay public awareness.

Two questions concerning education were identified as separate elements that do not contribute to any existing scale. These inquiries highlighted varying perspectives among pharmacists regarding the possibility of overqualification resulting from their educational backgrounds relative to job requirements. Notably, approximately 40% of respondents either agreed or strongly agreed with the concept of educational overqualification. Conversely, nearly one-third expressed disagreement, while a smaller proportion, less than one-third, maintained a neutral stance.

The fourth item of the Pharmacist Professional Perception Scale—‘Regulations in pharmacy are satisfied’—was intended to assess respondents’ satisfaction with the regulatory frameworks governing pharmacy practice in their respective countries, rather than to evaluate compliance with existing regulations. The mean score of 2.91 (SD 1.12) on this item reflects moderate dissatisfaction with the prevailing regulatory environment as perceived by participating pharmacists. It is acknowledged, however, that the phrasing of this item is grammatically imprecise and may have been interpreted differently by different respondents, representing a limitation of the instrument that should be addressed in future iterations of the scale.

Among a subgroup of specialized participants (*n* = 345), analysis revealed that 30.9% acknowledged a positive correlation between specialization and increased income. Conversely, 43.5% of pharmacists reported no apparent financial benefit associated with their specialisation, while 25.6% expressed ambivalence about this issue. The mean score for this question was 2.71 (1.4).

### 3.3. Associations Between Sociodemographic Factors and Outcomes

Associations between sociodemographic characteristics and the two outcome scales were examined using the Mann–Whitney U test for comparisons between two groups and the Kruskal–Wallis test for comparisons involving three or more groups, as detailed in Section 2.4. The study showed that various socio-demographic factors were linked to both the Job Satisfaction and Pharmacist Professional Perception Scales (*p* < 0.05). Specifically, workplace environment, monthly income, and employment status were associated with both scales (*p* < 0.05). Gender was only linked to the Job Satisfaction scale (*p* = 0.024). Geographical location, years of professional experience, age, and specialisation were connected to the Pharmacist Professional Perception Scale (*p* < 0.05). Results are presented in Table 4.

Female participants, those with a PhD, and employed in independent community pharmacies or industry settings (as opposed to medium to large chain community pharmacies or hospital pharmacies), as well as individuals with higher monthly incomes and those working part-time, exhibited greater job satisfaction (Table 4).

**Figure 1:**
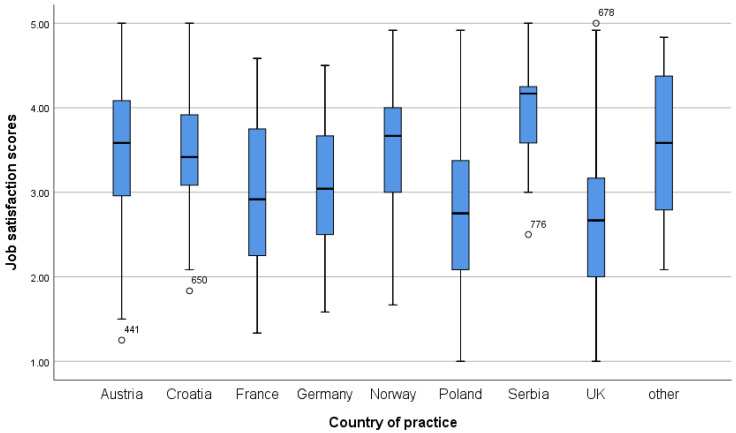
Job Satisfaction scores across different countries.

**Figure 2:**
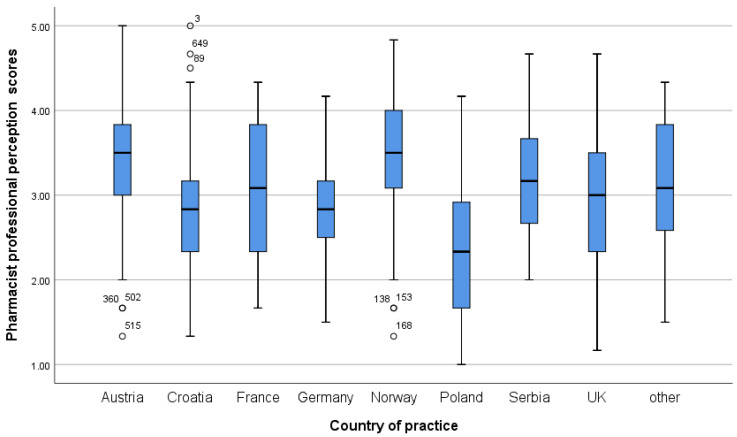
Pharmacists’ professional perception scores across different countries.

**Figure 3:**
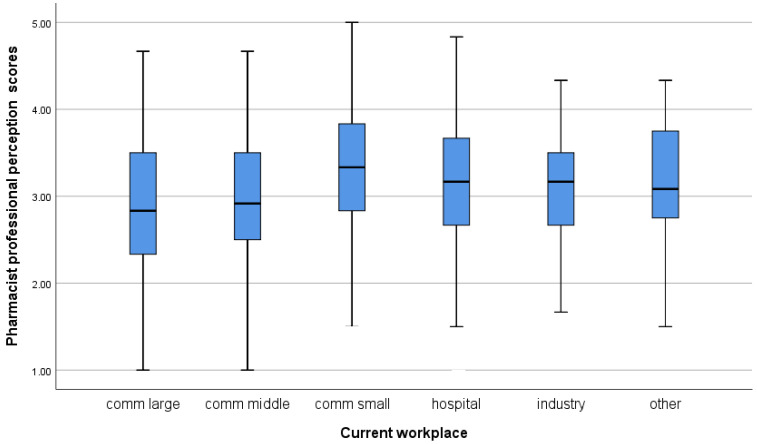
Pharmacist professional perception score depending on participants’ workplace.

**Figure 4:**
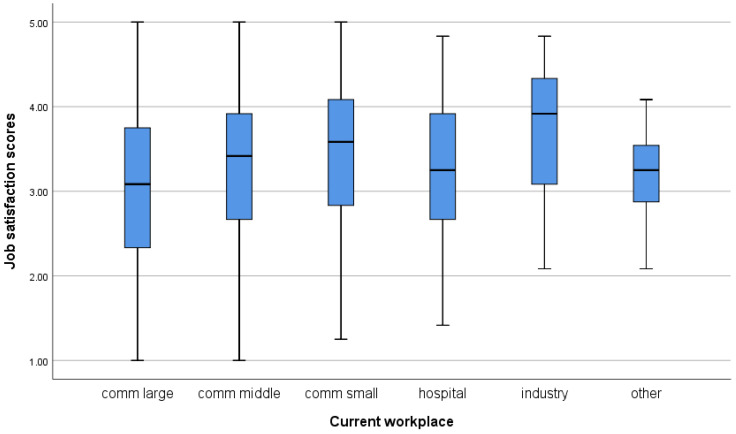
Job satisfaction scores depend on participants’ workplace.

Regarding the Pharmacist Professional Perception Scale, participants from Western European countries with longer work experience, who are older, hold a PhD and/or specialisation, work in independent community pharmacies, earn higher monthly incomes, and work part-time show a more favourable perception of the pharmacy profession (Table 4). A more detailed distribution of scores on both scales, by country and workplace, is shown in the boxplots in Figure 1, Figure 2, Figure 3 and Figure 4.

## 4. Discussion

The current study provides an exploratory evaluation of job satisfaction and professional perception among pharmacists from 17 European countries included in this multi-country convenience sample. The item-level distribution of job satisfaction scores observed in this study aligns closely with Herzberg’s two-factor theory of motivation (1959) [14], which distinguishes between hygiene factors—extrinsic elements such as salary, staffing adequacy, and physical working conditions whose absence generates dissatisfaction—and motivator factors—intrinsic elements such as collegial relationships, professional recognition, and inherent job interest that drive positive satisfaction. Consistent with this theoretical framework, the lowest-scoring items in the present study correspond predominantly to hygiene dimensions (staffing adequacy, mean 2.72; salary appropriateness, mean 2.98), whilst the highest-scoring items reflect motivator dimensions (collegial connection, mean 3.84; temporal engagement, mean 3.77). This distinction has important practical implications: improving hygiene factors may prevent active dissatisfaction, but enhancing motivator factors—through expanded clinical roles, greater professional autonomy, and structured recognition mechanisms—is likely necessary to generate meaningful improvements in professional fulfilment [15].

The findings reveal notable differences in satisfaction levels, emphasising the role of professional and environmental influences on pharmacists’ experiences. The overall average satisfaction score was 3.26 (SD 0.88), with the highest satisfaction linked to the sense of connection with colleagues (average 3.84, SD 1.08) and the lowest related to staff adequacy (average 2.72, SD 1.38).

Our findings regarding the impact of workplace environment, salary, and professional recognition on job satisfaction are consistent with those reported by Berassa [16], who highlighted similar influences across different healthcare settings. High workload and ethical tensions in high-volume community pharmacy settings have also been identified as important contributors to dissatisfaction [6]. Additionally, our study delves deeper into the issue of educational alignment, with roughly 40% of respondents feeling overqualified for their roles. This problem of perceived overqualification has been less frequently reported in other regions. Still, it is echoed in the study by Meilianti [10] which discussed global trends in pharmacist job satisfaction and highlighted discrepancies between job demands and educational levels.

In this sample, female pharmacists reported higher levels of job satisfaction than male pharmacists. This contrasts with Al-Jumaili et al. [7], where gender did not significantly affect satisfaction outcomes. This finding is also inconsistent with Carvajal and Popovici [17], who reported mixed gender effects on pharmacist job satisfaction in European settings. Such divergence may stem from differences in cultural and systemic factors across workplace environments, suggesting that gender roles and expectations may influence job satisfaction differently.

It is also important to note that the demographic profile of our sample—characterised by a median age of 40 years and a median work experience of 13.5 years—may not be fully representative of the pharmacy workforce in the participating countries. The web-based recruitment method may have preferentially engaged younger, more digitally active, and more organisationally affiliated pharmacists, and the perspectives of more senior practitioners may therefore be underrepresented in the data. This should be borne in mind when considering the implications of findings related to age and experience as predictors of satisfaction and professional perception.

The findings of this study indicate considerable variability in pharmacists’ job satisfaction and professional perception among pharmacists across different European countries. The observed differences do not follow a clear or easily interpretable pattern, making it difficult to explain why certain countries rank higher or lower on the job satisfaction scale. This heterogeneity suggests that job satisfaction or professional perception among pharmacists is influenced by a complex interplay of factors, including healthcare system organization, professional roles, workload, remuneration, and sociocultural context, which may vary substantially between countries.

Despite the absence of a consistent country-specific pattern, a broader comparison reveals a notable regional trend. Although these regional comparisons indicate consistent differences between Western and Eastern Europe, they depend on a basic categorization of countries. As a result, they should be viewed with caution, acknowledging the considerable diversity of healthcare systems and pharmacy practices within each region. For example, pharmacy practice in the United Kingdom differs markedly from that in France or Germany in terms of regulatory frameworks, scope of prescribing, and professional identity, despite all three countries being classified within the same Western European category. Similarly, the Eastern European grouping encompasses countries with widely varying healthcare system structures and economic contexts. Future research should adopt country-level analytical approaches to generate more nuanced comparative findings. In this sample, respondents from Western European countries reported higher professional perception scores, and a similar trend was seen for job satisfaction. However, these comparisons should be viewed with caution due to uneven country representation and simplified regional groupings. These results align broadly with previous studies indicating that healthcare system features and professional environments can affect pharmacists’ job satisfaction [9,18]. Structural differences in pharmacy workforce conditions across Europe have been documented by the World Health Organization, which noted that variations in professional autonomy, economic context, and scope of practice substantially shape pharmacist experiences [18,19]. Nevertheless, our data do not permit definitive conclusions about regional patterns across Europe, and the differences observed might also stem from sample composition and specific country factors not included in this research. The persistence of core dissatisfiers—particularly staffing inadequacy and salary—across settings is consistent with earlier foundational studies [19,20], suggesting these challenges remain structurally entrenched rather than episodic.

The link between specialised training and higher job satisfaction, as well as between higher income and greater job satisfaction, indicates that professional development and sufficient remuneration are vital for improving work conditions. This is supported by recent research by Ibrahim et al. [5], which found that career advancement opportunities were strongly associated with job satisfaction among pharmacists. Improving access to continuing education and offering clear pathways for career progression could therefore be essential strategies for healthcare leaders and policymakers aiming to enhance pharmacist retention and job satisfaction.

To further investigate the observed geographical differences in job satisfaction, future research should examine the specific systemic and policy factors that underlie these disparities. Understanding these underlying causes will be essential for developing effective interventions that improve professional fulfilment and retention within the pharmacy workforce.

A further contextual factor not captured in the present analysis is the degree to which extended professional pharmacy services—including medication review, pharmacist prescribing, and clinical consultation—are formally developed and recognised within each participating country’s healthcare system. The level of professional service development may constitute an important structural determinant of both job satisfaction and professional perception, as pharmacists practising within more developed service frameworks may experience greater professional autonomy, role clarity, and perceived recognition. Future studies should incorporate country-level indicators of pharmacy service development—such as those reported by the Pharmaceutical Group of the European Union (PGEU) [21] or the International Pharmaceutical Federation (FIP) [22,23]—as contextual covariates to enable more nuanced cross-national comparisons.

Acquiring knowledge about job satisfaction enables employers to address employees’ needs, reduce staff turnover, and enhance the work environment. The primary determinants of job satisfaction are intrinsic aspects of the job itself; that is, individuals are content with their work, yet they face challenges such as heavy workloads, inadequate salaries, and low respect [8]. Within the limits of this multi-country convenience sample, the present findings may provide preliminary signals about such challenges and potential areas for improvement. These findings can significantly aid pharmacy leadership, administration, and employers, but should not be interpreted as definitive evidence for the wider European pharmacy workforce. These findings may help inform future discussions among pharmacy leaders, employers, and policymakers to take proactive measures to enhance the quality of pharmaceutical care services. Developing and implementing a well-structured system that fosters a conducive working environment, appropriate remuneration, and greater autonomy may improve both job and career satisfaction.

## 5. Limitations

This study has several limitations. First, the web-based, convenience sampling method employed through EPhEU and partner organisations resulted in an unknown sampling frame. Because it was impossible to determine how many pharmacists received or accessed the invitation, calculating an accurate response rate was infeasible, and the sample’s representativeness cannot be formally assessed. Additionally, respondents were unevenly distributed across countries, with a higher concentration from certain settings, which further limits the generalisability of the findings to the entire European pharmacy workforce. The distribution of respondents across the 17 participating countries was markedly uneven. Austria contributed 34.9% of all responses. This distributional imbalance substantially limits the credibility of cross-national and regional comparisons. Consequently, the results should be viewed as reflecting only those pharmacists who opted to participate. Conducting the survey in English across multiple countries may also have reduced participation among pharmacists with limited English skills and introduced language-related response bias, further affecting representativeness. Readers should therefore interpret all findings as reflecting the perspectives of those pharmacists who voluntarily chose to participate, rather than as representative of European pharmacists as a whole.

Second, the data are self-reported, which may lead to response bias and non-response bias. Third, given the web-based distribution format, the sample may have inadvertently skewed towards younger and less experienced pharmacists, as well as those with greater digital engagement and stronger affiliations with professional organisations. The median work experience of 13.5 years should be compared with available workforce data for the participating countries to assess this potential bias, and we acknowledge that such comparisons were not possible within the scope of the present study.

Furthermore, the cross-sectional, single time-point design of the study means that the data represent a contemporaneous snapshot of respondents’ job satisfaction and professional perception as experienced during the survey window (October 2023–January 2024), rather than a stable longitudinal assessment. Satisfaction scores may therefore be influenced by transient situational or contextual factors present during this specific period, and caution is warranted in treating these findings as reflective of enduring professional attitudes.

Lastly, the cross-sectional design prevents any causal conclusions about the relationships between sociodemographic factors, job satisfaction, and professional perception. Future studies should focus on specific systemic and policy factors influencing geographical differences in job satisfaction and professional perception, preferably utilising more representative sampling methods and longitudinal research. Additionally, the monthly income variable was collected as net income should be reported, and no adjustment was made for cost-of-living or purchasing power parity differences across the 17 participating countries. Given the substantial variation in price levels and wage structures across the participating countries, direct cross-national income comparisons and the interpretation of income-satisfaction associations are subject to meaningful measurement imprecision. Finally, country-level structural variables—such as the degree to which extended professional pharmacy services are formally developed and legislatively supported—were not included as covariates in the analytical framework. Such variables may represent important contextual determinants of the observed cross-national variation in satisfaction and professional perception scores, and their omission limits the depth of interpretation that can be applied to the comparative findings.

## 6. Conclusions

This study provides preliminary, exploratory insights into the nature of job satisfaction and professional perception among pharmacists in 17 European countries, highlighting the potential role of both individual factors—such as educational background and employment status—and systemic factors—such as workplace setting, remuneration, and geographical context—in shaping professional experiences. Given the convenience sampling strategy and the methodological limitations described in the Limitations section, these findings should be regarded as hypothesis-generating rather than definitive. They may nonetheless serve as a useful foundation for future research employing more representative sampling methods and longitudinal designs, with the ultimate aim of developing evidence-based strategies to support pharmacist workforce retention and professional fulfilment across diverse European healthcare contexts.

## Figures and Tables

**Table 1:** Participants’ sociodemographic characteristics.

Variable	Category	Value (N, %)
Age	Median (range)	40 (20–75) years
Gender	Female	632 (80.1%)
	Male	150 (19.0%)
	Prefer not to say	7 (0.9%)
Current country of employment	Austria	275 (34.9%)
	Croatia	106 (13.4%)
	France	22 (2.8%)
	Germany	38 (4.8%)
	Norway	91 (11.5%)
	Poland	123 (15.6%)
	Serbia	29 (3.7%)
	United Kingdom	89 (11.3%)
	Other	16 (2.0%)
Current workplace	Community pharmacy (large chain)	274 (34.7%)
	Community pharmacy (small chain)	96 (12.2%)
	Community pharmacy (independent)	291 (36.9%)
	Hospital	96 (12.2%)
	Industry	21 (2.6%)
	Other	13 (1.6%)
Type of workplace ownership	Public/state	148 (18.8%)
	Private	641 (81.2%)
Current employment status	Full-time	495 (62.7%)
	Part-time	294 (37.3%)
Work experience	Median (IQR)	13.5 (6–22) years
Personal monthly income	Less than 1000 euros	67 (8.5%)
	1000–2000 euros	273 (34.6%)
	2000–3000 euros	256 (32.4%)
	More than 3000 euros	193 (24.5%)
Highest degree of education	Bachelor	91 (11.5%)
	Master	534 (67.7%)
	PharmD	83 (10.5%)
	PhD	40 (5.1%)
	Other	41 (5.2%)
Pharmacy specialization	With specialization	345 (43.7%)
	No specialization	444 (56.3%)

IQR—interquartile range.

**Table 2:** Participants job satisfaction scale scores.

Factor	Mean Score (Scale 1–5)	SD
I am often satisfied with my job	3.34	1.154
I leave work feeling I do something I enjoy	3.33	1.199
The time goes by quickly while I am at work	3.77	1.088
My work is valued	3.27	1.228
My salary is appropriate	2.98	1.291
My talents are fully utilised in my job	2.83	1.238
Knowing what I know now, if I had to decide all over again, I would still choose pharmacy	3.00	1.505
I am satisfied with my working conditions	3.08	1.229
I have independence in my work	3.42	1.210
I feel connected to my coworkers	3.84	1.079
Staffing is adequate; enough employees are hired to cover the workload in the pharmacy	2.72	1.378
There is a suitable work environment (such as space, ventilation, lighting, and hygiene facilities)	3.57	1.288
Job Satisfaction Scale	3.26	0.875

**Table 3:** Participants Pharmacist Professional Perception Scale scores.

Factor	Mean Score (Scale 1–5)	SD
Pharmacists enjoy public respect	3.16	1.238
Pharmacists are an essential part of the health system	4.36	1.020
The health system I work in recognises pharmacists’ competencies and supports pharmacists	2.74	1.212
Regulations in pharmacy are satisfied	2.91	1.124
Nurses and doctors are cooperative with pharmacists in questions related to therapeutic problems	2.90	1.122
A lay person (non-pharmacy profession person) is knowledgeable about the level of education of pharmacists	2.40	1.088
Pharmacist Professional Perception Scale	3.08	0.807

**Table 4:** Association between participants’ characteristics and job satisfaction and professional perception.

Variable	Category	Job Satisfaction Scale (Mean ± SD)	*p*	Pharmacist Professional Perception Scale (Mean ± SD)	*p*
Gender	Female	3.31 ± 0.84	0.024	3.10 ± 0.79	0.303
	Male	3.11 ± 0.98		3.01 ± 0.86	
Highest degree of education	Bachelor	3.11 ± 0.94	0.038	3.23 ± 0.81	0.049
	Master	3.27 ± 0.87		3.05 ± 0.81	
	PharmD	3.30 ± 0.91		3.10 ± 0.83	
	PhD	3.62 ± 0.76		3.33 ± 0.77	
Geographical location ^a^	Western Europe	3.30 ± 0.86	0.103	3.29 ± 0.71	<0.001
	Eastern Europe	3.20 ± 0.91		2.67 ± 0.82	
Workplace ^b^	Pharmacy (large)	3.06 ± 0.89	<0.001	2.86 ± 0.82	<0.001
	Pharmacy (small)	3.31 ± 0.91		2.96 ± 0.79	
	Pharmacy (independent)	3.42 ± 0.84		3.31 ± 0.76	
	Hospital	3.22 ± 0.83		3.13 ± 0.79	
	Industry	-		3.11 ± 0.69	
Years of work experience	<5 years	3.29 ± 0.86	0.066	2.99 ± 0.92	0.027
	5–20 years	3.21 ± 0.86		3.05 ± 0.80	
	>20 years	3.37 ± 0.89		3.20 ± 0.74	
Age	<30 years	3.22 ± 0.89	0.102	2.93 ± 0.88	0.002
	31–45 years	3.22 ± 0.86		3.04 ± 0.81	
	46–55 years	3.40 ± 0.88		3.22 ± 0.73	
	>55 years	3.27 ± 0.89		3.23 ± 0.74	
Monthly income	<2000 euros	3.18 ± 0.87	0.011	2.84 ± 0.85	<0.001
	>2000 euros	3.33 ± 0.87		3.27 ± 0.72	
Type of workplace ownership	State/public	3.29 ± 0.86	0.817	3.12 ± 0.78	0.430
	Private	3.26 ± 0.88		3.07 ± 0.81	
Employment status	Full-time	3.21 ± 0.90	0.046	2.93 ± 0.85	<0.001
	Part-time	3.35 ± 0.82		3.34 ± 0.66	
Specialization	Yes	3.32 ± 0.85	0.164	3.17 ± 0.75	0.005
	No	3.22 ± 0.90		3.01 ± 0.84	

*p*-values were acquired using the Mann-Whitney test for comparisons between two groups, or the Kruskal-Wallis test for comparisons involving three or more groups. ^a^ Western European countries: Austria, France, Germany, Malta, Norway, Portugal, Switzerland, UK; Eastern European countries: Bosnia and Herzegovina, Bulgaria, Croatia, Kosovo, Montenegro, Poland, Romania, Serbia, Turkey. ^b^ Independent pharmacy (1 community pharmacy); small chain (2–4 pharmacies); large chain (>5 pharmacies).

## Data Availability

The data presented in this study are available on request from the corresponding author due to privacy and ethical restrictions.
